# Layer groups: Brillouin-zone and crystallographic databases on the Bilbao Crystallographic Server

**DOI:** 10.1107/S205327332100783X

**Published:** 2021-09-24

**Authors:** Gemma de la Flor, Bernd Souvignier, Gotzon Madariaga, Mois I. Aroyo

**Affiliations:** a Institute of Applied Geosciences, Karlsruhe Institute of Technology, Karlsruhe, Germany; b Institute for Mathematics, Astrophysics and Particle Physics, Radboud University Nijmegen, The Netherlands; c Departamento de Física, Universidad del País Vasco UPV/EHU, Spain

**Keywords:** Bilbao Crystallographic Server, layer groups, Brillouin-zone database

## Abstract

The section of the Bilbao Crystallographic Server (https://www.cryst.ehu.es/) dedicated to subperiodic groups contains crystallographic and Brillouin-zone databases for the layer groups. The crystallographic databases include the generators/general positions (*GENPOS*), Wyckoff positions (*WYCKPOS*) and maximal subgroups (*MAXSUB*). The Brillouin-zone database (*LKVEC*) offers **k**-vector tables and Brillouin-zone figures of all 80 layer groups which form the background of the classification of their irreducible representations. The symmetry properties of the wavevectors are described applying the so-called *reciprocal-space-group* approach and this classification scheme is compared with that of Litvin & Wike [(1991), *Character Tables and Compatibility Relations of the Eighty Layer Groups and Seventeen Plane Groups*. New York: Plenum Press]. The specification of independent parameter ranges of **k** vectors in the representation domains of the Brillouin zones provides a solution to the problems of uniqueness and completeness of layer-group representations. The Brillouin-zone figures and **k**-vector tables are described in detail and illustrated by several examples.

## Abstract 2

The set of databases of the Bilbao Crystallographic Server (https://www.cryst.ehu.es/) including crystallographic data on generators, general positions, Wyckoff positions, maximal subgroups and Brillouin-zone figures and **k**-vector tables for all 80 layer groups are discussed in detail and illustrated.

## 1. Introduction   

The Bilbao Crystallographic Server (https://www.cryst.ehu.es/) (Aroyo *et al.*, 2006 ▸, 2011 ▸; Tasci *et al.*, 2012 ▸) is a free web site that grants access to specialized databases and tools for the resolution of different types of crystallographic, structural chemistry and solid-state physics problems. The server has been operating for more than 20 years, and is in constant improvement and development, offering free-of-charge tools to study an increasing number of crystallographic systems (Elcoro *et al.*, 2017 ▸; Gallego *et al.*, 2019 ▸; de la Flor *et al.*, 2019 ▸). The programs on the server are organized into different sections depending on their degree of complexity, in such a way that the more complex tools make use of the results obtained by the simpler ones. The Bilbao Crystallographic Server (hereafter referred to as BCS) is built on a core of databases that include data from *International Tables for Crystallography*, Vol. A, *Space-group Symmetry* (Aroyo, 2016 ▸; henceforth abbreviated as ITA), Vol. A1, *Symmetry Relations between Space Groups* (Wondratschek & Müller, 2010 ▸), and Vol. E, *Subperiodic Groups* (Kopský & Litvin, 2010 ▸; henceforth referred to as ITE). A **k**-vector database with Brillouin-zone figures and classification tables of the wavevectors for all 230 space groups (Aroyo *et al.*, 2014 ▸) is also available in the server, together with the magnetic and double-space-group databases. The magnetic and the incommensurate structure databases are also hosted on the server.

Aside from the subgroups of space groups with three-dimensional lattices which are again space groups, there also exist subgroups called *subperiodic groups* with translation lattices of dimensions one or two. These groups are essential to describe polymers, nanotubes, nanowires, layered and multilayered materials. The interest in materials with sub­periodic symmetry is constantly growing due to their outstanding properties and possible technological applications. There are three types of subperiodic groups: *frieze* groups (two-dimensional groups with one-dimensional translation lattices), *rod* groups (three-dimensional groups with one-dimensional translation lattices) and *layer* groups (three-dimensional groups with two-dimensional translation lattices). The crystallographic data for subperiodic groups are compiled in ITE and also now offered online in the BCS section dedicated to subperiodic groups. This section includes programs which give access to the generators/general positions (*GENPOS*), Wyckoff positions (*WYCKPOS*) and maximal subgroups (*MAXSUB*) databases.

In addition, we have developed the Brillouin-zone database of layer groups which contains **k**-vector tables and Brillouin-zone figures that form the background of the classification of the irreducible representations of layer groups. The Brillouin-zone figures and the wavevector data for space groups are well established and many authors have contributed to the standardization of the data (see *e.g.* Miller & Love, 1967 ▸; Bradley & Cracknell, 1972 ▸; Cracknell *et al.*, 1979 ▸; Stokes & Hatch, 1988 ▸). For layer groups, however, the Brillouin-zone and wavevector descriptions proposed by several authors (Ipatova & Kitaev, 1985 ▸; Hatch & Stokes, 1986 ▸; Milošević *et al.*, 2008 ▸) are incomplete and difficult to compare due to the lack of standards in the classification and nomenclature of the **k** vectors. To the best of our knowledge, the only complete compilation of layer-group **k** vectors together with the Brillouin-zone diagrams is found in the work of Litvin & Wike (1991 ▸) (in the following, referred to as LW). In this description the **k** vectors are labelled following the classification scheme of space-group **k** vectors used by Cracknell *et al.* (1979 ▸). Based on the group–subgroup relations between space and layer groups and using the so-called *reciprocal-space-group* approach (*cf.* Wintgen, 1941 ▸; Aroyo & Wondratschek, 1995 ▸; Aroyo *et al.*, 2014 ▸), we have derived the **k**-vector data and Brillouin-zone figures for all 80 layer groups and compared them with the classification of LW. An auxiliary tool for the complete characterization of wavevectors is also available: given the wavevector coefficients referred to a primitive or conventional dual basis, the program assigns the corresponding wavevector symmetry type, specifies its LW label, determines the layer little co-group of the wavevector and generates the arms of the wavevector star. The BCS Brillouin-zone database of layer groups is accessed by the retrieval tool *LKVEC*.

The aim of this contribution is to present the crystallographic databases and the Brillouin-zone database for layer groups available in the BCS. In the following, the retrieval tools *GENPOS*, *WYCKPOS*, *MAXSUB* and *LKVEC*, and the procedure to derive and classify the **k** vectors of layer groups are described in detail. The utility of the programs is demonstrated by several illustrative examples.

## 2. Crystallographic databases for layer groups   

The BCS section *Subperiodic Groups: Layer, Rod and Frieze Groups* hosts the layer-group crystallographic databases. The structure of these databases is similar to that of the space groups – they include information on generators, general positions, Wyckoff positions and maximal subgroups for the 80 layer groups. Apart from the data shown in ITE the server offers additional information and computer tools that allow the generation of data not available in ITE. The BCS programs and databases use the so-called *standard* or *default* settings of the layer groups. These are the specific settings of layer groups that coincide with the *conventional* layer-group descriptions found in ITE. For layer groups with more than one description in ITE, the following settings are chosen as standard: (i) *cell-choice* 1 description for the two monoclinic/oblique layer groups *p*11*a* (No. 5) and 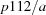 (No. 7) described with respect to three cell choices in ITE, and (ii) *origin choice* 2 descriptions (*i.e.* origin at an inversion centre) for the three layer groups 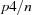 (No. 52), 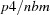 (No. 62) and 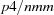 (No. 64) listed with respect to two origins in ITE.

Note that, following the conventions of ITE, the *ab* plane is the plane of periodicity for the layer groups and therefore the translation vectors are of the form 

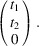




### 2.1. Generators and general positions   

The BCS database of layer groups includes the list of generators/general positions of each layer group. These data can be retrieved using the program *GENPOS*, by specifying the sequential ITE layer-group number (which, if unknown, can be determined by choosing the corresponding Hermann–Mauguin symbol from a table with the layer-group symbols). The generators and/or general positions of layer groups are specified by their coordinate triplets, the matrix-column representations of the corresponding symmetry operations and their geometric interpretation:

(*a*) The list of coordinate triplets (*x*, *y*, *z*) reproduces the data of the *General Positions* blocks of layer groups found in ITE. The coordinate triplets may also be interpreted as shorthand descriptions of the matrix presentation of the corresponding symmetry operations.

(*b*) Matrix-column presentation of symmetry operations. With reference to a coordinate system, consisting of an origin *O* and a basis (**a**_1_, **a**_2_, **a**_3_), the symmetry operations of layer groups are described by (3 × 4) matrix-column pairs.

(*c*) Geometric interpretation. The geometric interpretation of symmetry operations is given (i) following the conventions in ITE [including the symbol of the symmetry operation, its glide or screw component (if relevant) and the location of the related symmetry element], and (ii) using the Seitz notation. It is worth pointing out that, in contrast to the online and printed editions of ITE, the programs of BCS use the standard International Union of Crystallography Seitz notation. For example, a twofold rotation around the *c* axis is denoted by 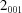 instead of 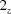 (for details, *cf.* Litvin & Kopský, 2014 ▸).

**Figure 1:**
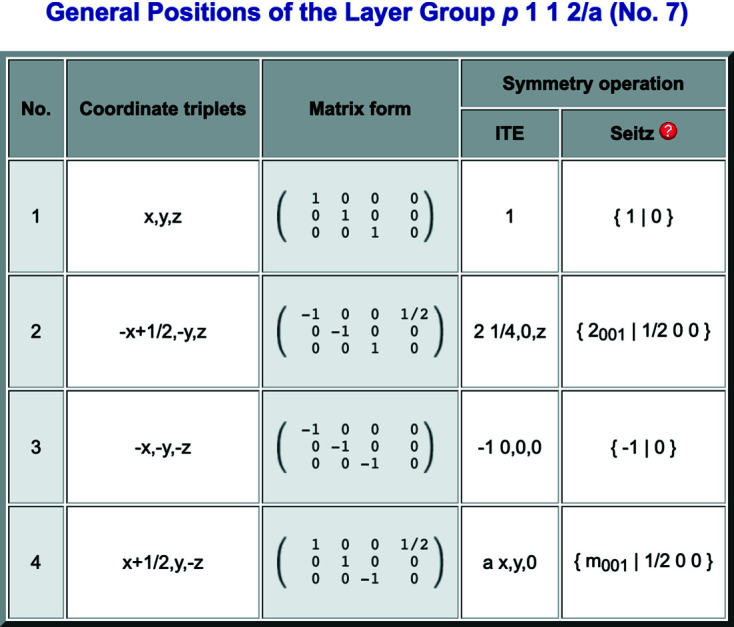
General-position table of the layer group 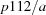 (No. 7), provided by the program *GENPOS*.

Fig. 1 ▸ shows the general position for the layer group 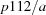 (No. 7) in the default setting (*cell-choice* 1).

The program *GENPOS* lists the generators and/or general positions of layer groups in the *standard/default setting* as well as in conventional non-standard settings of monoclinic/rectangular and orthorhombic/rectangular layer groups described in Table 1.2.6.1 of ITE (option *ITE settings*). In addition, the program can produce the data in any non-conventional setting if the transformation relating the *non-conventional setting* to the *standard* one is specified (option *Non-conventional setting*). The matrix-column pair (**P**, **p**) of the transformation relating the two *settings* consists of two parts: a linear part **P** defined by a (3 × 3) matrix, which describes the change of direction and/or length of the basis vectors (**a**_1_, **a**_2_, **a**_3_)_non-conv_ = (**a**_1_, **a**_2_, **a**_3_)_stan_ **P**, and an origin shift **p** = 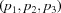 defined as a (3 × 1) column, whose coefficients describe the position of the non-conventional origin with respect to the standard one.

The URL of the program *GENPOS* is https://www.cryst.ehu.es/subperiodic/get_sub_gen.html.

### 2.2. Wyckoff positions   

**Figure 2:**
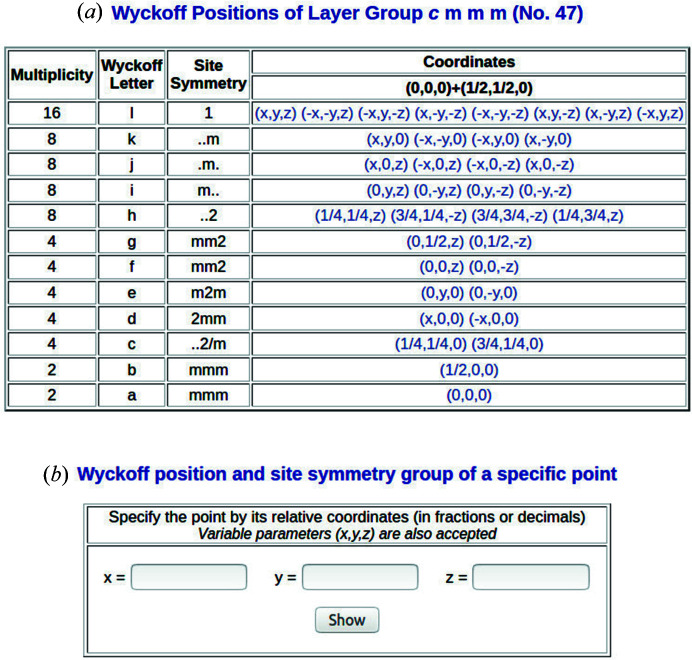
A screenshot of the output of the program *WYCKPOS*, showing (*a*) the Wyckoff positions of the layer group *cmmm* (No. 47), and (*b*) the interface of the auxiliary tool for the determination of the Wyckoff position and the site-symmetry group of a specific point.

**Figure 3:**
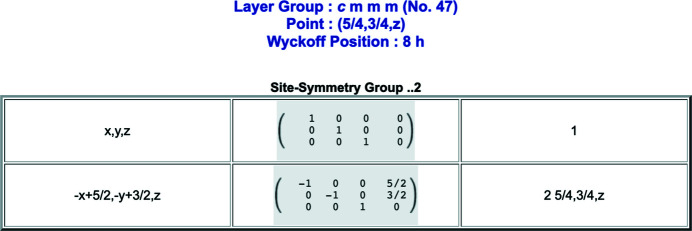
Output of the auxiliary tool in *WYCKPOS* that shows the symmetry operations of the site-symmetry group for points with coordinates 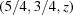 of the layer group *cmmm* (No. 47). The points belong to the Wyckoff position 8*h* with site-symmetry group ..2.

The BCS Wyckoff-positions database for layer groups is accessible under the *WYCKPOS* program. The data on Wyckoff positions can be retrieved by specifying the ITE number of the layer-group type. As a result, the program *WYCKPOS* shows a table with the Wyckoff positions [see Fig. 2 ▸(*a*)]. Following ITE, each Wyckoff position is characterized by its multiplicity, Wyckoff letter, site-symmetry group and a set of coordinate triplets of the Wyckoff-position points in the unit cell. For centred subperiodic groups, the centring translations are listed above the coordinate triplets. The site-symmetry groups [see column three of Fig. 2 ▸(*a*)] are described by oriented symbols which display the same sequence of symmetry directions as the layer-group symbols (*cf.* Table 1.2.4.1 of ITE). An explicit listing of the symmetry operations of the site-symmetry group of a point is obtained by clicking directly on its coordinate triplet. A recently implemented auxiliary tool permits the identification of the Wyckoff position and the site-symmetry operations of a point specified by its coordinate triplet [see Fig. 2 ▸(*b*)]. The program accepts as input relative point coordinates in fractions, decimals or variable parameters (indicating a generic value). Fig. 3 ▸ shows the list of the symmetry operations of the site-symmetry group of the layer group *cmmm* (No. 47) for the points 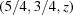 . The points belong to the Wyckoff position 8*h* with site-symmetry group ..2.

Apart from the *standard/default setting* option the program is also able to calculate the Wyckoff positions in different ITE (conventional) settings (option *ITE settings*) or with respect to a *non-conventional setting* if the corresponding coordinate transformation (**P**, **p**) is defined (option *Non-conventional setting*).

The URL of the program *WYCKPOS* is https://www.cryst.ehu.es/subperiodic/get_sub_wp.html.

### 2.3. Maximal subgroups   

**Figure 4:**
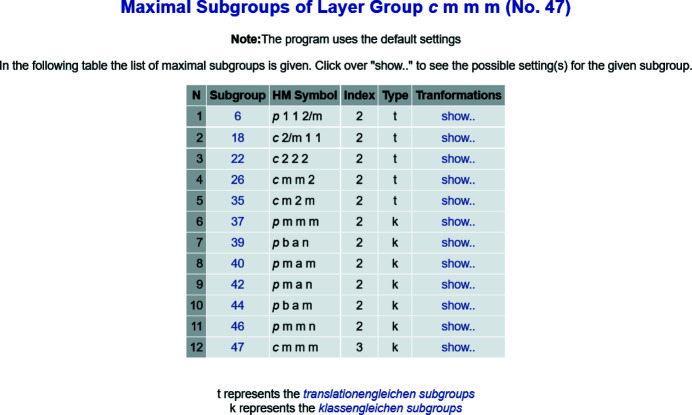
Maximal subgroups of the layer group *cmmm* (No. 47) provided by the program *MAXSUB*.

The listing of maximal subgroups of layer groups available in ITE is incomplete and lacks additional information, such as, for example, possible unit-cell transformations and/or origin shifts involved. In contrast, the BCS database of maximal subgroups of layer groups provides the complete listing (not just by type but individually) of (i) all maximal non-isotypic subgroups for each layer group, and (ii) all maximal isotypic subgroups of indices 2, 3 and 4. The list of maximal subgroups is retrieved by the access tool *MAXSUB*. Each subgroup is specified by its ITE number, Hermann–Mauguin symbol, index, subgroup type (t for *translationengleiche* or k for *klassengleiche*) and transformation matrix-column pair (**P**, **p**) that relates the standard setting of the group with that of the subgroup (see Fig. 4 ▸). The different maximal subgroups are distributed into conjugacy classes. The identification of the subgroup symmetry operations as a subset of the elements of the group is achieved by an optional tool of *MAXSUB* that transforms the general-position representatives of the subgroup to the coordinate system of the group.

The URL of the program *MAXSUB* is https://www.cryst.ehu.es/subperiodic/get_sub_maxsub.html.

## 3. Classification of wavevectors   

A layer group 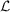 is defined as a three-dimensional crystallographic group with periodicity restricted to a two-dimensional subspace. Just as for space groups, the general strategy for determining the irreducible representations, called irreps for short, of such a group is to exploit the fact that it contains the translation subgroup 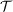 as a normal abelian subgroup and that irreps of abelian groups are one-dimensional. The elements of 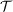 have matrix-column pairs of the form 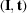 where 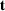 lies in a two-dimensional lattice 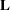 with basis 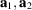 . Each one-dimensional irrep of 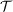 is then of the form 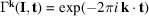 for a 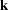 vector 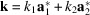 in the *reciprocal space* spanned by the *reciprocal basis* 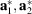 defined by 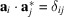 .

Starting from an irrep 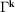 of 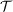 , an irrep of the full layer group 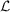 is obtained by first extending 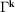 to the stabilizer of 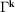 under the conjugation action of 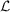 and then inducing to the full group 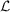 . When the induced irrep of 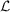 is restricted to 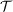 , it is the sum of those one-dimensional irreps 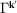 of 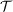 which lie in the orbit of 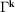 under the conjugation action of 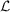 and these are precisely the irreps of 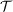 that yield irreps of 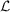 equivalent to the one obtained from 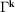 . For the action of 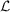 on the 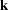 vectors only the part acting on the plane of periodicity of 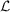 is relevant and the restriction of 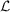 to this plane gives rise to a plane group 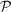 associated to 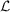 . In the convention of ITE, the matrix-column pairs of 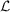 are expressed with respect to a basis 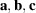 such that the first two basis vectors span the plane of periodicity. Therefore, the matrices of the linear parts of the plane group 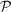 are simply the upper 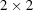 diagonal blocks of the linear parts of the layer group 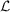 .

An explicit computation shows that the 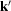 in the orbit of 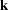 are of the form 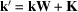 for 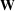 an element of the point group 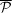 of 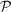 and 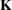 an element from the *reciprocal lattice* 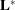 of 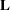 , *i.e.* for 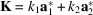 with 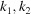 integers. The set of elements 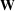 for which 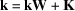 is called the *little co-group* 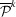 of 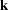 . The **k** vector is called *general* if 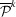 contains only the identity element of 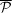 , *i.e.* 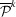 = {*I*}; otherwise 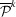 > {*I*} and **k** is *special* (Bradley & Cracknell, 1972 ▸; Dresselhaus *et al.*, 2008 ▸). If { 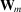 } is the set of coset representatives of the decomposition of 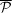 with respect to 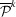 , then the set of 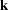 vectors { 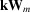 } is called the *star* of 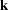 while the vectors 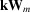 are called the *arms of the star*. Using the relation between the elements of the layer group 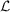 and the plane group 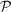 associated to 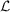 , in analogy to 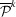 , one defines the *little layer co-group* 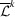 which is essential for the derivation of the irreps of 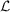 .

The preceding discussion indicates the importance of the so-called *reciprocal plane group* 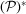 , defined as the symmorphic plane group having the reciprocal lattice 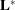 as translation vectors and 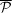 as point group. It is crucial that the operations in 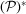 act on reciprocal space, *i.e.* they act on rows, as opposed to 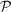 which acts on columns. In order to identify 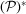 with one of the symmorphic plane groups in their standard setting, the action on reciprocal space (on rows) has to be transformed into one on direct space (on columns).

### 3.1. Transformation between reciprocal space and direct space   

After fixing a basis 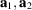 for direct space, the action of the point group 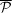 of 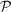 on 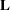 is given by 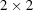 matrices 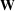 acting on columns. We now have to relate the action on the reciprocal lattice 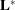 to that on 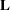 . Defining

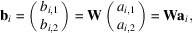

the basis 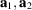 is mapped by 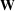 to the new basis 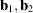 . The corresponding matrix 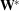 for the action on reciprocal space (on rows) must then map the reciprocal basis 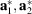 to the reciprocal basis 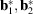 of 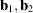 . From 

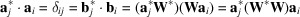

it follows that 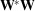 must be the identity matrix 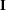 . We thus have 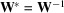 , *i.e.* the action on the rows of reciprocal space is given by the inverse matrices acting on the columns of direct space. Since 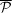 is a group, it contains with every matrix 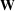 of course also the inverse matrix 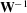 ; hence the set of matrices remains the same, but they now act from the right on rows.

As an example, we take the layer group 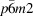 (No. 78). Restricting the action to the plane of periodicity gives the plane group 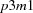 (No. 14). If we look at the 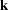 vector 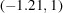 , applying the threefold rotation with matrix

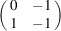

to the row 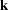 gives 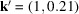 and applying it again gives 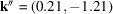 . On the other hand, applying the reflection with reflection line 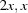 and matrix 

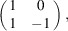

we see that the row 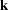 is mapped to 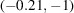 , which is equal to 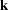 up to a reciprocal-lattice vector. The *star* of 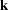 consisting of the 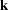 vectors in the orbit under 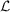 is therefore 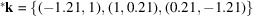 .

As we have just demonstrated, it is natural to consider the action of the reciprocal plane group 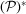 on rows, but on the other hand 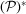 is by construction isomorphic to a symmorphic plane group 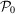 . In order to identify 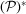 with the proper symmorphic plane group, the action on rows has to be transformed to an action on columns. This is of course achieved by simply transposing the matrices of the point group 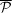 , since 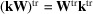 and the transpose 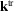 of a row is a column. The symmorphic plane group 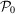 isomorphic to 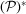 is therefore the group 

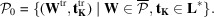

The only problem that may occur is that 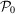 is not given in its standard setting. This can be seen from different (strongly interrelated) perspectives:

(i) The reciprocal basis 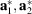 may not be a conventional basis of the lattice 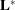 . For example, the reciprocal lattice of a hexagonal lattice is also a hexagonal lattice, but while the vectors 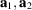 in the conventional basis have an angle of 120°, the reciprocal basis vectors 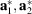 enclose an angle of 60° and are therefore not a conventional basis of a hexagonal lattice.

(ii) The matrices 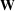 of the point group fix the metric tensor 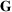 of the lattice 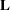 , *i.e.* one has 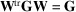 . The transposed matrices fix the inverse metric tensor 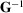 as can be seen from inverting the above equation: 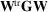 = 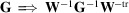 = 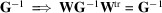 . This corresponds to the fact that the reciprocal lattice 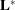 has metric tensor 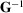 . But 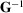 may not be the metric tensor of a lattice with respect to its conventional basis. For example, a hexagonal lattice has a metric tensor of the form 

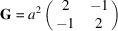

which has inverse 

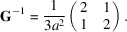

This is the metric tensor of a hexagonal lattice with basis vectors having an angle of 60°, *i.e.* in a non-conventional setting.

(iii) The transposed matrices 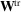 simply do not occur as matrices of one of the point groups of plane groups in their standard setting. For example, the transposed matrix 

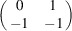

of the matrix

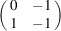

of a threefold rotation 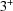 does not belong to the point group of any of the plane groups.

For plane groups, the hexagonal lattice is actually the only case in which the transposed matrices do not belong to a point group in its standard setting. For all other lattices, the inverse of the metric tensor still belongs to a conventional basis or, in other words, the set of matrices of the point group does not change by transposing.

In order to identify the symmorphic plane group 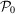 in the case of a hexagonal lattice, the group has to be transformed to a basis in the conventional setting, *i.e.* which has a metric tensor equal to a multiple of

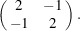

Such a transformation is 

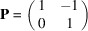

(or its compositions with powers of a sixfold rotation) and the transposed matrices of the point group have to be conjugated by this. This interchanges the two types of reflections (in *p*6*mm*), those with normals along the two basis vectors and their sum and those with reflection lines along the basis vectors (and their sum). For example, for 

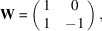

the reflection with reflection line 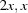 , one gets 

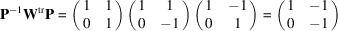

which is the reflection with reflection line along the *a* axis.

As a result, for a layer group with plane group of type *p*3*m*1, the symmorphic plane group 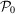 isomorphic to the reciprocal plane group 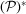 is of type *p*31*m* and vice versa.

Based on the identification of the reciprocal plane group 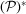 with a symmorphic plane group 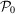 , the special 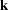 vectors for a layer group 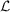 can be directly read off from the Wyckoff positions of 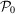 .

### 3.2. Crystallographic conventions in the classification of layer-group irreps   

The isomorphism between a reciprocal plane group 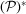 and a symmorphic plane group 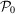 allows the application of crystallographic conventions in the classification of the wavevectors (and henceforth of the irreps) of the layer groups 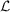 :

(i) The unit cells of the symmorphic plane groups listed in ITA can replace the Brillouin zones as unit cells of the reciprocal space. To find all irreps of 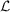 , it is necessary to consider only the wavevectors of the so-called *representation domain*. It is defined as a simply connected part of the (first) Brillouin zone (a unit cell of the reciprocal space) which contains exactly one **k** vector of each orbit of **k**. The asymmetric units of plane groups can serve as representation domains. The advantage of choosing the crystallographic unit cells and their asymmetric units becomes evident in layer groups where the Brillouin zones may belong to different topological types depending on the ratios of the lattice parameters. Lines on the Brillouin zone may appear or disappear or change their relative sizes depending on the lattice parameters. In contrast to that, the unit cells and their asymmetric units of ITA are independent of the ratios of the lattice parameters.

(ii) The action of the reciprocal plane group 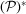 on the wavevectors results in their distribution into orbits of symmetry-equivalent **k** vectors with respect to 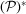 . Thanks to the isomorphism of ( 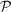 ) 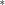 with the symmorphic plane group 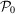 , the different types of **k** vectors correspond to the different kinds of point orbits (Wyckoff positions) of 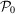 . In this way, a complete list of the special sites in the Brillouin zone of ( 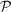 ) 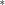 is provided by the Wyckoff positions of 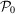 found in ITA. The site-symmetry groups of ITA correspond to the little co-groups of the wavevectors and the number of arms of the star of a wavevector follows from the multiplicity of the Wyckoff position. The Wyckoff positions with zero, one and two variable parameters correspond to special **k**-vector points, special **k**-vector lines and special (or general) **k**-vector planes, respectively. A **k**-vector type, *i.e.* the set of all **k** vectors corresponding to a Wyckoff position, consists of complete orbits of **k** vectors and thus of full stars of **k** vectors. The different orbits (and stars) of a **k**-vector type are obtained by varying the free parameters. Correspondingly, the irreps of **k** vectors of a **k**-vector type are interrelated by parameter variation and are said to belong to the same type of irreps (Boyle, 1986 ▸). In this way all wavevector stars giving rise to the same type of irreps are related to the same Wyckoff position and designated by the same Wyckoff letter.

(iii) A complete set of irreps of 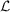 is derived by considering exactly one **k**-vector representative per **k**-vector orbit. To achieve that, it is necessary to specify the exact parameter ranges of the independent **k**-vector regions within the representation domain (or the asymmetric unit). While such data are not available in the literature, the Brillouin-zone database of BCS offers the listing of the exact parameter ranges for the **k** vectors which are absolutely necessary for the solution of the problems of uniqueness and completeness of layer-group irreps. For this purpose it is advantageous to describe the different **k**-vector stars belonging to a Wyckoff position applying the so-called *uni-arm description*. Two **k** vectors of a Wyckoff position are called uni-arm if one can be obtained from the other by parameter variation. The description of **k**-vector stars of a Wyckoff position is called uni-arm if the **k** vectors representing these stars are uni-arm. Frequently, in order to achieve a uni-arm description, it is necessary to transform **k** vectors to equivalent ones. In addition, to enable a uni-arm description, symmetry lines outside the asymmetric unit may be selected as orbit representatives. Such a segment of a line is called a *flagpole*. Examples of flagpoles are displayed in Fig. 9, and Figs. 10 and 11 for the layer group *cm*2*m* (No. 35) [*cf.* Section 5.2 for a detailed account of the use of flagpoles in the uni-arm description of **k**-vector types that belong to Wyckoff position 2*a* of the reciprocal plane group 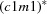 ].

## 4. The Brillouin-zone database for layer groups   

The **k**-vector data of the Brillouin-zone database of the BCS are accessed by the retrieval tool *LKVEC* which uses as input the ITE number of the layer group. The output consists essentially of wavevector tables and figures. There are several sets of figures and tables for the same layer group when its Brillouin-zone shape depends on the lattice parameters of the reciprocal lattice. The **k**-vector data are the same for layer groups of the same arithmetic crystal class.

In the **k**-vector tables, the wavevector data of LW are compared with the Wyckoff-position data of ITA. In the figures, the Brillouin zones and the representation domains of LW, and the asymmetric units, chosen often in analogy to those of ITA, are displayed.

LW describe the monoclinic/rectangular layer groups with respect to a setting that is different from the conventional one found in ITE. Using the relationship between the two settings, we have transformed the **k**-vector data of LW to the conventional setting of ITE for all six monoclinic/rectangular arithmetic crystal classes: 211*p*, 211*c*, *m*11*p*, *m*11*c*, 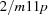 and 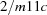 . The transformed special **k**-vector points, lines and planes keep the LW labels of the **k** vectors from which they were derived.

The URL of the program *LKVEC* is https://www.cryst.ehu.es/subperiodic/get_layer_kvec.html.

### 4.1. Guide to the tables   

Each **k**-vector table is headed by the corresponding Hermann–Mauguin symbol of the layer group, its ITE number and the symbol of the arithmetic crystal class to which the layer group belongs. If there is more than one table for an arithmetic crystal class, then these tables refer to different geometric conditions for the lattice parameters that are indicated after the symbol of the arithmetic crystal class. The set of layer groups of the arithmetic crystal class are also indicated in the headline block. They are followed by the symbol of the corresponding reciprocal plane-group type together with the conditions for the lattice parameters of the reciprocal lattice, if any (asterisks denote reciprocal-space quantities). From the **k**-vector table there is a link to the corresponding Brillouin-zone figure.

The **k**-vector tables consist of two parts: (i) ‘Litvin & Wike’ description and (ii) ‘Plane-group description’. The first three columns under the heading ‘Litvin & Wike’ refer to the description of the **k**-vectors found in Tables 24 and 25 of LW. It consists of labels of **k**-vectors (column 1), their parameter descriptions (column 2) and their layer little co-group (column 3 for primitive lattices and column 4 for *c*-centred). Note that LW substitutes the Greek-character labels for the symmetry points and lines inside the Brillouin zone by a symbol consisting of two Roman characters, *e.g.* GM instead of Γ, LD instead of Λ *etc*. In order to enable the uni-arm description new **k**-vector types, equivalent to those of LW, are added to the **k**-vector lists. Equivalent **k** vectors (related by the sign ∼) are designated by the same labels; additional indices distinguish the new **k** vectors.

Different **k** vectors with the *same* LW label always belong to the same **k**-vector type, *i.e.* they correspond to the same Wyckoff position. **k** Vectors with *different* LW labels may either belong to the same or to different types of **k** vectors. When **k** vectors with different LW labels belong to the same **k**-vector type, the corresponding parameter descriptions are followed by the letters ‘ex’ (from Latin, with the meaning of ‘from’ or ‘out of’). Symmetry points or lines of symmetry of LW, related to the same Wyckoff position, are grouped together in a block. In the **k**-vector tables, neighbouring Wyckoff-position blocks are distinguished by a slight difference in the background colour. The parameter description of the uni-arm region of a **k**-vector type is shown in the last row of the corresponding Wyckoff-position block.

The wavevector coefficients of LW (column 2 of the **k**-vector tables) refer always to a primitive basis irrespective of whether the conventional description of the group in ITE is with respect to a centred or primitive basis. For that reason, for layer groups with centred lattices, the wavevector coefficients with respect to the usual conventional reciprocal basis, *i.e.* dual to the conventional centred basis, are listed in the column under the heading ‘Conventional’ of the **k**-vector tables. The relations between the conventional coefficients (*k*_1_, *k*_2_) and the primitive coefficients ( 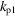 , 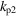 ) are summarized in Table 1 ▸. (For layer groups with primitive lattices, the wavevector coefficients referred to a primitive basis coincide with those referred to the basis dual to the conventional one of ITA.)

The layer little co-group data of each **k** vector are listed under the heading ‘Layer little co-group’ of the **k**-vector tables. The layer little co-groups are subgroups of the point group of the layer group and are described by *oriented point-group* symbols (as is customary for site-symmetry groups of Wyckoff positions).

**Figure 5:**
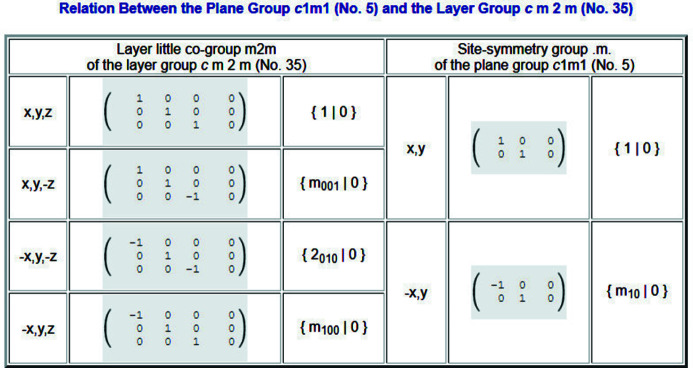
Relation between the symmetry operations of the site-symmetry group .*m*. of the plane group 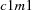 (No. 5) and the layer little co-group 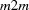 of the layer group 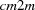 (No. 35) for the **k** vector DT = (−*u*, *u*).

The data for the crystallographic classification scheme of the wavevectors are listed under the heading ‘Plane-group description’ in the **k**-vector tables. The columns ‘Wyckoff positions’ show the ‘multiplicity’, ‘Wyckoff letter’ and ‘site-symmetry’ of the Wyckoff positions of the corresponding symmorphic plane group 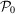 of ITA which is isomorphic to the reciprocal plane group ( 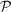 ) 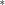 . The multiplicity of a Wyckoff position divided by the number of lattice points in the conventional unit cell of ITA is equal to the number of arms of the star of the corresponding **k** vector. The alphabetical sequence of the Wyckoff positions determines the sequence of the LW labels. Unlike in ITA, the tables start with the Wyckoff letter ‘*a*’ for the Wyckoff position of the highest site symmetry. Site symmetries are described by means of oriented point-group symbols which are also links to more detailed information on the symmetry operations of the site-symmetry group. Besides the shorthand description, the matrix-column representation and the geometric representation of the symmetry operations of the site-symmetry group, the program also provides a table with the relationship between the symmetry operations of the site-symmetry group and the layer little co-group (*cf.* Fig. 5 ▸ and Section 3.1 for detailed explanations).

The *parameter description* of the Wyckoff positions is shown in the last column of the wavevector tables under the heading ‘Coordinates’. It consists of a representative coordinate doublet of the Wyckoff position and algebraic statements for the description of the independent parameter range. In some cases, the algebraic expressions are substituted by the designation of the parameter region in order to avoid clumsy notation. Because of the dependence of the shape of the Brillouin zone on the lattice parameter relations there may be vertices of the Brillouin zone with a variable coordinate. If such a point is displayed and designated in the tables and figures by an upper-case letter, then the label of its variable coefficient used in the parameter-range descriptions is the same letter but typed in lower case.

Because of the isomorphism between 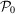 and ( 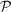 ) 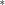 the coordinate doublets of the Wyckoff positions of 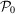 can be interpreted as **k**-vector coefficients ( 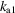 , 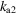 ) determined with respect to the conventional ITA basis of 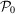 . The relation between the ITA coefficients ( 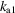 , 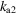 ) and the conventional coefficients ( 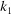 , 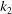 ) is shown in Table 2 ▸.

**Figure 6:**
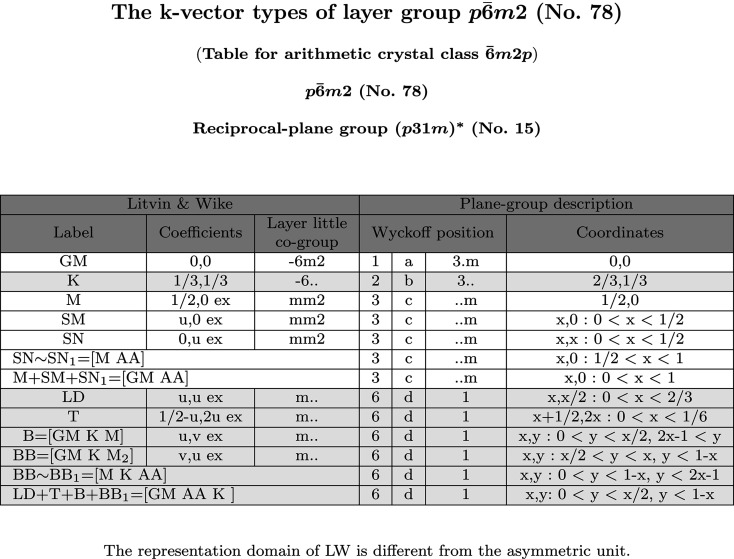
**k**-Vector table of the layer group 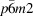 (No. 78) as shown on the BCS. The Brillouin-zone diagram is shown in Fig. 8.

At the bottom of the web page with the **k**-vector table one finds an auxiliary tool which allows the complete characterization of a wavevector of the reciprocal space (not restricted to the first Brillouin zone): given the **k**-vector coefficients referred either to a primitive (LW) or to a conventional basis, the program assigns the **k** vector to the corresponding wavevector symmetry type, specifies its LW label, and calculates the layer little co-group and the arms of the **k**-vector star. Consider again the example of the **k** vector with coefficients 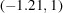 for the layer group 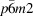 (No. 78), *cf.* Fig. 6 ▸ and Fig. 8. It is a vector outside the first Brillouin zone and its coefficients do not correspond to any of the parameter descriptions of the **k**-vector representatives listed in Fig. 6 ▸. The output of the auxiliary tool indicates that **k** 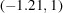 is a point of a special **k**-vector line of type SM and belongs to the Wyckoff-position block 3*c*. As already commented in Section 3.1, its star consists of three **k** vectors, 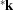 = {(−1.21, 1), (1, 0.21), (0.21, −1.21)}. The site-symmetry group ..*m* is generated by a reflection plane that can be identified by direct inspection among the symmetry operations of (*p*31*m*) 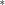 . The layer little co-group, however, is *mm*2, due to the additional reflection in the layer plane.

### 4.2. Guide to the figures   

The headline of each Brillouin-zone figure includes the same information as the **k**-vector tables: the Hermann–Mauguin symbol of the layer group, the ITE number and the symbol of the arithmetic crystal class to which the layer group belongs. Different figures for the same arithmetic crystal class are distinguished by the geometric conditions for the lattice. The corresponding conditions for the lattice parameters of the reciprocal lattice are indicated after the symbol of the reciprocal plane group.

The Brillouin zones are two-dimensional objects in the reciprocal space. The coordinate axes are designated by *k_x_* and *k_y_*, and the origin with coefficients (0, 0) always coincides with the centre of the Brillouin zone and is called Γ (indicated as GM in the **k**-vector tables). In the Brillouin-zone figures the representation domains of LW are compared with the asymmetric units of ITA. A statement of whether the representation domain of LW and the asymmetric unit are identical or not is given below the **k**-vector table. The asymmetric units are often *not* fully contained in the Brillouin zone, but protrude from it, in particular by flagpoles.

**Figure 7:**
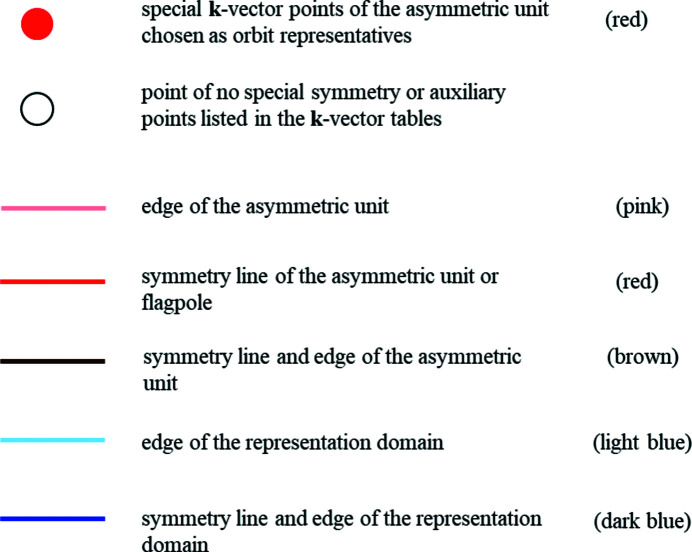
Colour coding of points and lines applied in the Brillouin-zone diagrams.

The representatives of the orbits of **k**-vector symmetry points or **k**-vector symmetry lines, as well as the edges of the representation domains of LW and of the asymmetric units are brought out in colour (see Fig. 7 ▸):

(*a*) *Symmetry points*. A representative point of each orbit of special **k**-vector points is designated by a circle filled in red with its label also in red. Note that a point is coloured red only if it is really a special point, *i.e.* a point whose layer little co-group is a supergroup of the little co-groups of the points in its neighbourhood. In the figures, a point is marked by its label and an empty circle if it is listed in the corresponding **k**-vector table but is not a point of special symmetry. For example, points listed by LW are not coloured if they form part of a symmetry line or a symmetry plane. The same designation is used for the auxiliary points that have been added in order to facilitate the comparison between the traditional and the reciprocal plane-group descriptions of the **k**-vector types.

(*b*) *Symmetry lines*. A line is coloured in red with its label also in red only if it is a special **k**-vector line, *i.e.* the layer little co-groups of the points on the line are supergroups of the little co-groups of the points in its neighbourhood. The colour of the line is pink for an edge of the asymmetric unit which is not a symmetry line. The colour of the line is brown with the name in red for a line which is a symmetry line as well as an edge of the asymmetric unit. The edges of the representation domains are coloured light blue if the representation domain of LW does not coincide with the asymmetric unit. Edges of the representation domain and their labels are coloured dark blue if they are symmetry lines. Flagpoles are always coloured in red. Coordinate axes, edges of the Brillouin zone or auxiliary lines are displayed by thin solid black lines.

## 5. Examples   

The relation between the traditional and the reciprocal group descriptions of the wavevector types is illustrated by the following examples. The figures and tables included here form part of the output of the access tool *LKVEC*.

### 5.1. **k**-Vector table and Brillouin zone for the layer group *p* 62*m* (No. 78)   

**Figure 8:**
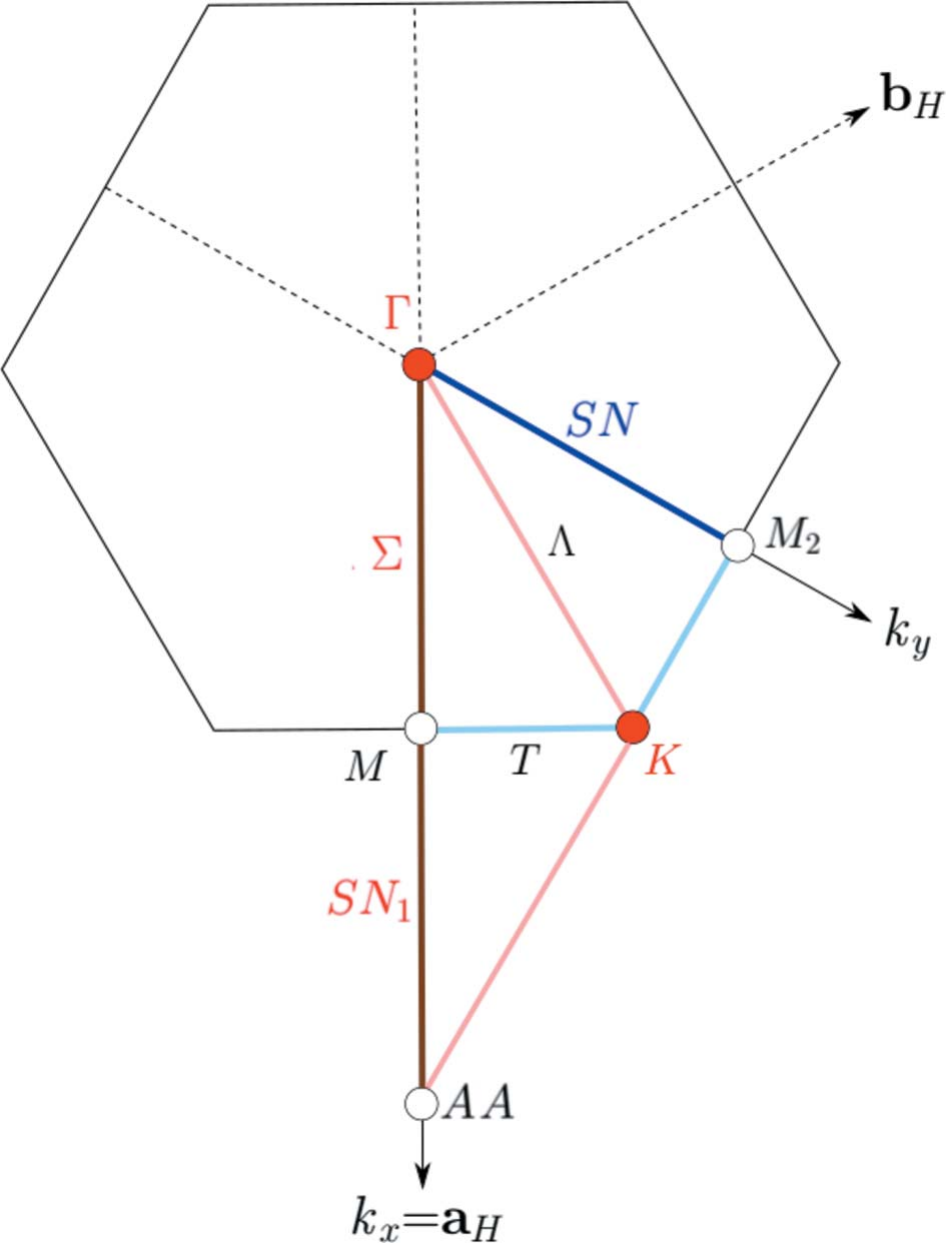
Brillouin zone and representation domain of LW, and asymmetric unit of the layer group 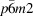 (No. 78). The reciprocal plane group is 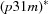 (No. 15). The representation domain of LW is different from the asymmetric unit.

The **k**-vector table and the Brillouin-zone diagram of the hexagonal layer group 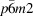 (No. 78) are shown in Fig. 6 ▸ and Fig. 8 ▸, respectively. The reciprocal lattice of a hexagonal *p* lattice is also a hexagonal *p* lattice and the Brillouin zone is a hexagon. The conventional basis for the reciprocal lattice has 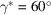 while the ITA description of hexagonal layer groups is based on a basis **a**_H_, **b**_H_ with 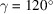 . In the Brillouin-zone diagrams, the axis **k**_*x*_ is taken along **a**_H_ while **k**_*y*_ points out in the direction of **a**_H_ + **b**_H_.

The **k** vectors of 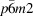 (No. 78) listed by LW (Fig. 6 ▸) are distributed in four **k**-vector types: (i) the Wyckoff-position block 1*a* formed by the GM point, (ii) the block 2*b* formed by the K point, (iii) the **k**-vector point M and the **k**-vector lines SM and SN correspond to the block 3*c*, and (iv) the Wyckoff-position block 6*d* formed by the **k**-vector lines LD and T and the **k**-vector planes B and BB. The parameter description of a **k**-vector type is given in the last column of the table. Consider for example the line SM which, according to the ITA description, forms part of the **k**-vector type that is assigned to the Wyckoff position 3*c* with a site-symmetry group ‘..*m*’. Its parameter description *x*, 0: 0 < *x* < 1/2 indicates that the independent segment of the line *x*, 0 in the asymmetric unit is limited by the special **k**-vector points Γ (*x* = 0) and M (*x* = 1/2) with *x* varying between 0 and 1/2. The parameter descriptions of the uni-arm regions of the **k**-vector types are shown in the last row of the corresponding Wyckoff-position block. For example, in the block for position 3*c*, the **k**-vector line SN is equivalent (by a threefold rotation) to 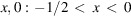 which in turn is equivalent (by a translation) to the line 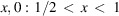 , denoted by SN_1_. This gives the uni-arm description M+SM+SN_1_ for the Wyckoff position 3*c* in Fig. 6 ▸.

As the asymmetric unit and the representation domain do not coincide, their edges are coloured in pink and light blue, respectively. It has already been pointed out that **k**-vector points and lines are brought out in red only if they are special **k**-vector points and lines. For example, the lines T and LD (indicated as Λ on Fig. 8 ▸) are not coloured as special lines since they belong to the **k**-vector type of the Wyckoff-position block 6*d*, and their symmetry coincides with that of the neighbouring points of the symmetry plane B = [GM K M]. The points GM (indicated as Γ on Fig. 8 ▸) and K, however, are represented by red circles as they are special **k**-vector points (*cf.* Fig. 6 ▸). Likewise, the line SM (indicated as Σ on Fig. 8 ▸) is coloured in brown because it is an edge of the asymmetric unit and at the same time is a special **k**-vector symmetry line. The **k**-vector line SN is coloured in dark blue as it is a special symmetry line along the edge of the representation domain. As already indicated, the **k**-vector line SN together with SM and the point M belong to the special **k**-vector type of the Wyckoff-position block 3*c*, *i.e.* all these different wavevectors belong to the same **k**-vector type. Although M is explicitly listed by LW as a special **k**-vector point, it is represented by an open circle in Fig. 8 ▸: in fact, it joins the symmetry lines SM and SN_1_ to a continuous line as its little co-group type coincides with those of the points on the lines.

### 5.2. **k**-Vector table and Brillouin zone for the layer group *cm*2*m* (No. 35)   

**Figure 9:**
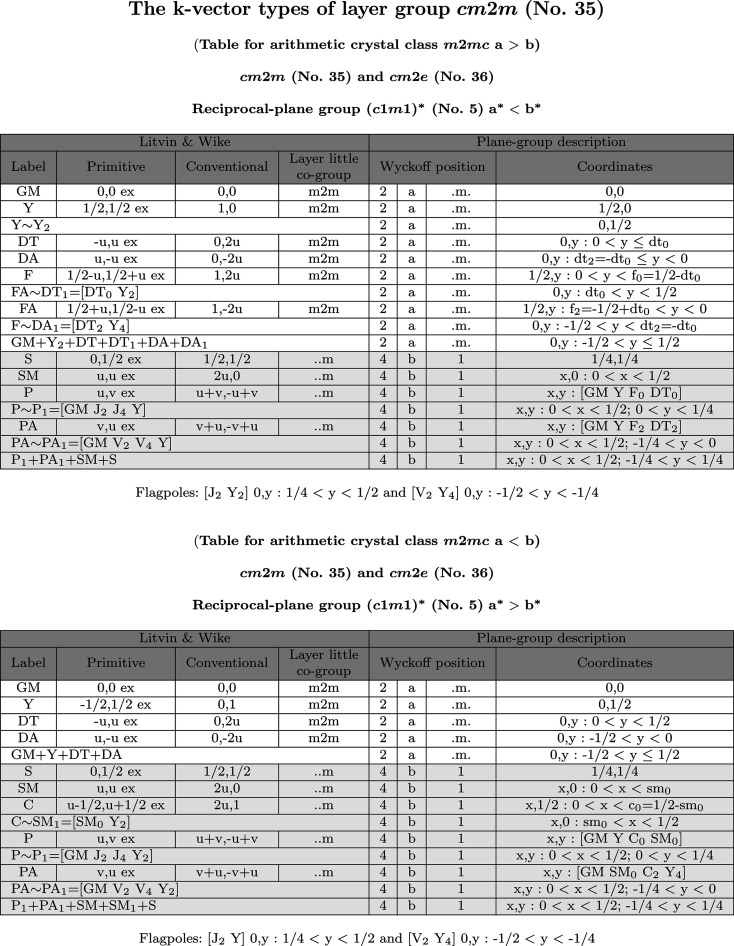
**k**-Vector tables of the layer groups with arithmetic crystal class *m*2*mc*, acute (*a* > *b*) and obtuse (*a* < *b*) cases, as shown on the BCS. The Brillouin-zone diagrams are shown in Figs. 10 ▸ and 11 ▸.

**Figure 10:**
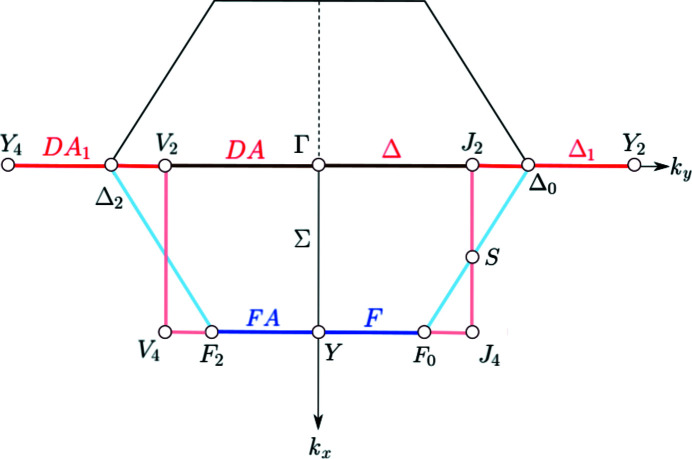
Brillouin zone and representation domain of LW, and asymmetric unit of the arithmetic crystal class *m*2*mc*: layer group *cm*2*m* (No. 35) and *cm*2*e* (No. 36) acute case (*a* > *b*). The reciprocal group is 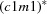 (No. 5). The representation domain of LW does not coincide with the asymmetric unit.

**Figure 11:**
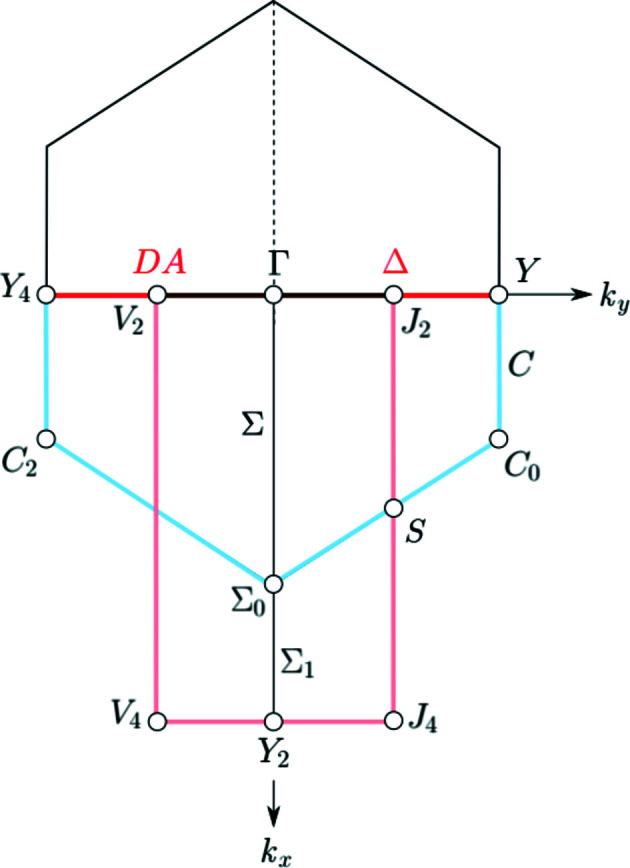
Brillouin zone and representation domain of LW, and asymmetric unit of the arithmetic crystal class *m*2*mc*: layer group *cm*2*m* (No. 35) and *cm*2*e* (No. 36) obtuse case (*a* < *b*). The reciprocal group is 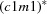 (No. 5). The representation domain of LW does not coincide with the asymmetric unit.

The layer group *cm*2*m* (No. 35) is an example of ortho­rhombic layer groups with a *c*-centred lattice. It belongs to the arithmetic crystal class *m*2*mc* which also includes the layer group *cm*2*e* (No. 36). The **k**-vector tables and Brillouin-zone figures for the layer groups belonging to the arithmetic crystal class *m*2*mc* are given in Fig. 9 ▸, and Figs. 10 ▸ and 11 ▸, respectively.

The **k**-vector tables show all special wavevectors with their coefficients and layer little co-groups as specified in Tables 24 and 25 of LW. The wavevector coefficients with respect to the conventional reciprocal basis, *i.e.* dual to the conventional centred basis, are listed in the column under the heading ‘Conventional’ of the **k**-vector tables. For example, a **k**-vector point of the DT line (Fig. 9 ▸) with primitive coefficients 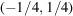 is described as 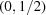 with respect to a basis dual to the conventional basis of *cm*2*m*.

The comparison of the wavevector list of LW and the reciprocal plane-group description indicates clearly the redundancy of most of the **k** vectors given by LW. In fact, for the derivation of a complete set of irreps it is necessary to consider just two **k**-vector types: a general one corresponding to the general Wyckoff position 4*b*, and the **k**-vector symmetry line related to the special Wyckoff position 2*a*. The large number of additional **k** vectors given in the tables of LW are due to two main reasons:

(*a*) The more symmetry a layer group has lost compared with its holosymmetric layer group (layer groups whose point groups are holohedral), the more **k** vectors are introduced in LW. In the case of *cm*2*m*, the holosymmetric layer group is *cmmm* (No. 47), and the lines DA and FA are examples of such additional **k** vectors. In most cases these additional **k** vectors can be avoided by extending the parameter range in the **k**-vector space.

(*b*) In the transition from a holosymmetric 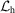 to a non-holosymmetric layer group 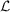 , the order of the little co-group of a special **k** vector in 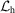 may be reduced in 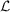 and, as a result, the special **k** vector in 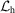 may lose its ‘special nature’ in 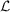 . Such **k** vectors become part of a more general **k**-vector type (*i.e.* assigned to a Wyckoff position of lower site symmetry) and can be described by an extension of the corresponding parameter range. Consider, for example, the **k**-vector points Γ and Y in the tables of *cm*2*m*. They are special **k**-vector points lying on the reflection plane of 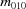 in the holosymmetric layer group *cmmm*, but in (the non-holosymmetric group) *cm*2*m* which does not contain 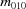 the two points form part of the special **k**-vector line with the uni-arm description: 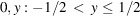 (*cf.* the last row of the 2*a* Wyckoff-position block, Fig. 9 ▸).

The Brillouin zone of the layer groups of the arithmetic crystal class *m*2*mc* is a non-regular hexagon (*cf.* Figs. 10 ▸ and 11 ▸). Depending on the relation between the lattice parameters *a* and *b*, two topologically different Brillouin zones are to be distinguished: (i) the *acute* case with 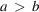 and (ii) the *obtuse* case with 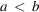 . Because of the reflection 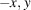 (with normal 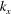 ) of the reciprocal plane group 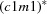 , the representation domain is only one half of the hexagon: for example, in the acute case (Fig. 10 ▸) it is the trapezium with vertices 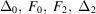 (light blue boundary). The asymmetric unit is different from the representation domain: it is the rectangle with vertices 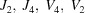 (pink boundary), *i.e.* the points 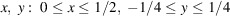 . While the representation domains of the acute and obtuse unit cells have the more complicated form of a trapezium, the asymmetric units in both cases have the topologically identical and relatively simple shape of a rectangle.

As already indicated, the points Γ, Y_2_ and S (acute case), and Γ, Y and S (obtuse case) are not special **k**-vector points but form part of special lines and planes and in the diagrams they are represented by open circles. The line SM is not a symmetry line and is represented by a thin black line because it is located inside the Brillouin zone. The lines DT and DA are coloured in brown because they are symmetry lines and at the same time are edges of the asymmetric unit. Parts of DT and DA are also coloured in red because they correspond to flagpoles. The **k**-vector lines F and FA (acute case) are coloured in dark blue as they are symmetry lines along the edges of the representation domain.

Because of the special shape of the Brillouin zone and the representation domain for the acute case ( 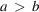 ), the special **k**-vector line corresponding to the Wyckoff-position block 2*a* splits into several segments: the lines DT and DA, located inside the Brillouin zone, and the lines F and FA (coloured dark blue) at the border of the Brillouin zone (*cf.* Figs. 9 ▸ and 10 ▸). For the description of the end points of the segments, it is necessary to introduce additional parameters as dt_0_ and f_0_ whose values depend on the specific relations between the lattice parameters. [In fact, the vertices 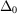 and F_0_ of the representation domain have the coordinates 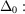 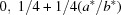 and F_0_: 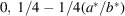 .] The use of flagpoles enables the uni-arm description: the flagpole [J_2_ Y_2_] 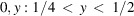 is equivalent to the segment [V_4_ Y] 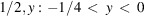 and the flagpole [V_2_ Y_4_] 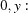 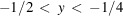 is equivalent to the segment [Y J_4_] 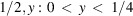 . The uni-arm description of the **k**-vector type of the Wyckoff position 2*a* is shown in the last row of the Wyckoff-position block and it is formed by the union of the points GM and Y_2_, the lines DT, DA, DT_1_(∼FA) and DA_1_ (∼F). Its parameter description (0, *y*) with *y* varying in the range (−1/2, 1/2) coincides with that of the acute case. The parameter description of the flagpole and its parameter range with respect to the basis of the reciprocal group are given below the **k**-vector table.

## 6. Conclusions   

In this paper we have presented the layer-group crystallographic and wavevector databases of the BCS, together with the programs which give access to these data. Like the rest of the programs on the server, these tools are freely available and can be accessed via user-friendly web interfaces. The programs *GENPOS*, *WYCKPOS* and *MAXSUB* provide access to generators/general positions, Wyckoff positions and maximal subgroup information, respectively.

The wavevector database which contains the Brillouin-zone figures and wavevector tables for all 80 layer groups was recently implemented on the BCS. One can access the database through the program *LKVEC*. In this compilation, the representation domains and lists of special **k** vectors in the tables on layer-group representations by Litvin & Wike (1991 ▸) are compared with the figures and wavevector data derived applying the reciprocal-space-group approach. This new database provides a solution to the completeness problem of layer-group representations by specifying the independent parameter ranges of general and special **k** vectors within the representation domains.

## Figures and Tables

**Table 1:** ‘Conventional’ **k**-vector coefficients {k}_{j} (*i.e.* with respect to a basis dual to the conventional basis of ITA) expressed by the ‘primitive’ **k**-vector coefficients {k}_{{\rm p}j} (*i.e.* referred to a primitive basis) for the different Bravais lattices in direct space

Bravais lattice	{k}_{1}	{k}_{2}
*mp*, *op*, *tp*, *hp*	{k}_{{\rm p}1}	{k}_{{\rm p}2}
*oc*	{k}_{{\rm p}1}+ {k}_{{\rm p}2}	-{k}_{{\rm p}1}+{k}_{{\rm p}2}

**Table 2:** ‘Conventional’ **k**-vector coefficients {k}_{j} (*i.e.* with respect to a basis dual to the conventional basis of ITA) expressed by the ITA **k**-vector coefficients {k}_{{\rm a}j} (*i.e.* referred to the conventional ITA basis of {\cal P}_{0}) for the different Bravais lattices in direct space

Bravais lattice	{k}_{1}	{k}_{2}
*mp*, *op*, *tp*	{k}_{{\rm a}1}	{k}_{{\rm a}2}
*oc*	{2k}_{{\rm a}1}	{2k}_{{\rm a}2}
*hp*	{k}_{{\rm a}1}-{k}_{{\rm a}2}	{k}_{{\rm a}2}
